# *CCNE1* amplification among metastatic sites in patients with gynecologic high-grade serous carcinoma

**DOI:** 10.1016/j.gore.2021.100850

**Published:** 2021-08-21

**Authors:** Benjamin Margolis, Fanny Dao, Michael Licciardi, Selim Misirlioglu, Narciso Olvera, Sitharam Ramaswami, Douglas A. Levine

**Affiliations:** a Laura and Isaac Perlmutter Comprehensive Cancer Center, NYU Langone Health, 550 1st Avenue, New York, NY 10016, USA; b Genome Technology Center, NYU Langone Health, 550 First Avenue, New York, NY 10016, USA

**Keywords:** Ovarian cancer, CCNE1, Copy number, Metastasis

## Abstract

**Objective:**

We sought to characterize the variability of *CCNE1* amplification among metastatic sites of *CCNE1* amplified high grade serous carcinoma (HGSC) cases to investigate the feasibility of targeting this alteration for therapeutic purposes.

**Methods:**

Patients with *CCNE1* amplified HGSC who underwent surgical cytoreduction with metastatic sites were identified from institutional molecular profiling reports and a population of HGSC cases screened using digital droplet PCR (ddPCR). Cases with normal *CCNE1* copy number were included as controls. Slides from metastatic sites were cut from formalin-fixed paraffin-embedded tissue blocks, dissected for tumor of > 50% purity, and underwent DNA extraction. *CCNE1* copy number was determined by ddPCR. Tumor purity was confirmed with mutant *TP53* allele fraction from targeted massively parallel sequencing.

**Results:**

Four of 15 patients from an institutional database screened by ddPCR were found to have *CCNE1* amplification. Three additional patients were identified from a query of institutional commercial clinical reports. Among these 7 *CCNE1* amplified cases (2 uterine, 5 ovarian), 5 showed preservation of *CCNE1* amplification (copy number > 5) among all metastatic sites. The remaining 2 cases had multiple metastatic sites without preserved *CCNE1* amplification. Non-amplified cases had predominantly normal *CCNE1* copy number across metastatic sites.

**Conclusions:**

*CCNE1* amplification is an early genomic event in HGSC and is preserved in most metastatic sites suggesting a uniform response to pathway targeting therapies.

## Highlights

• *CCNE1* amplification is conserved among metastatic sites in *CCNE1*-amplified high-grade serous carcinomas.
• Limited *CCNE1* copy number heterogeneity among *CCNE1*-amplified cases suggests some genomic change during metastasis.
• Digital droplet PCR can be used to quantify *CCNE1* copy number from archival specimens of high-grade serous carcinomas.

## 1 Introduction

Ovarian cancer is the second most common gynecologic malignancy in the United States, with an incidence of 10.2 cases per 100,000 susceptible people. (U.S. Cancer Statistics Working Group, 2019) Approximately 20% of high-grade serous carcinoma (HGSC), the most common subtype of ovarian cancer, have been found to harbor amplification of *CCNE1,* which codes for the cell cycle protein cyclin E. (Stronach et al., 2018) Patients with *CCNE1*-amplified HGSC have been shown to have a higher risk of chemoresistance to primary treatment and poor overall survival compared to those without *CCNE1* amplification. (Karst et al., 2014, Nakayama et al., 2010) *CCNE1* amplification leading to decreased survival and treatment resistance has also been shown in ovarian clear cell and endometrioid carcinomas. (Nakayama et al., 2016, Ayhan et al., 2017)

Cyclin E complexes with cyclin-dependent kinase 2 (CDK2) to facilitate entry into the S phase of the cell cycle to initiate DNA replication. (Moroy et al., 2004) Cyclin E is expressed during the G1/S transition and is also expressed when senescent cells are re-entering the cell cycle from a G0 state. *CCNE1* amplification has been found in a number of malignancies including sarcomas, non-small cell lung cancer, leukemia, lymphoma, breast and ovarian cancers. (Moroy et al., 2004) *CCNE1* amplification is thought to promote oncogenesis by promoting cell cycle re-entry and centrosome amplification. (Etemadmoghadam et al., 2010, Kuhn et al., 2016) Fluorescence in-situ hybridization (FISH) and immunohistochemistry (IHC) analysis have shown that *CCNE1* amplification is linked to high protein expression of cyclin E in 46–55% of cases. (Karst et al., 2014)

*CCNE1* amplification has been evaluated for its role as an initiating factor and driver mutation and subsequently as a therapeutic target. *CCNE1* amplification and increased cyclin E expression have both been shown in serous tubal intraepithelial carcinomas, the putative precursor lesion to HGSC. (Karst et al., 2014, Kuhn et al., 2016) Karst et al induced Cyclin E expression in p53 compromised fallopian tube epithelial cells and described accelerated cell growth, loss of contact inhibition and an absence of stress-induced apoptosis consistent with oncogenic transformation. Accordingly, *CCNE1* knock out by siRNA and shRNA has been shown to reduce cell viability in vitro. (Nakayama et al., 2010, Etemadmoghadam et al., 2010) The addition of *CCNE1* amplification to p53 mutations in HGSC is proposed to lead to oncogenesis by coupling an increased drive for replication with an inability to relate the G1-S transition. (Karst et al., 2014)

Several studies have examined the genomic relationship between the primary and metastatic sites of disease in HGSC. (Tsao et al., 1993, Marchion et al., 2013, Khalique et al., 2009, Brodsky et al., 2014) Although many alterations found in ovarian cancers are clonal, changes in the gene expression and mutational signatures at metastatic sites suggest that some adaptation may account for varied response to therapy.

We sought to evaluate the role of *CCNE1* amplification as an early driver event in oncogenesis by examining *CCNE1* copy number status in primary and metastatic sites in untreated newly diagnosed *CCNE1-*amplified HGSC at the time of initial surgery. Based on existing pre-clinical data showing that *CCNE1* amplification is present in HGSC precursor lesions, we hypothesize that *CCNE1* amplification will be a truncal event found across all sites of disease when amplified in the primary tumor. To utilize *CCNE1* amplification as a therapeutic target, it will be necessary to demonstrate that this event is consistently present across the entirety of the metastatic tumor burden.

## 2 Methods

### 2.1 Patient selection

FoundationOne CDx is an FDA-approved companion diagnostic from Foundation Medicine that detects mutations, copy number alterations and genomic signatures in patient tissues using a targeted capture next generation sequencing platform. Patients with *CCNE1* amplification of their primary tumor were identified by searching clinical reports of patients tested at our institution with the commercial Foundation Medicine platform between 2014 and 2019. Most patients with recurrent ovarian cancer had commercial testing during this time period. The electronic medical records of patients with documented *CCNE1* amplification were reviewed, and the availability of archival formalin-fixed paraffin-embedded (FFPE) specimens from metastatic disease sites was confirmed. Additional cases with *CCNE1* amplification were identified using an institutional cancer registry database of all high-grade serous malignancies from 2010 to 2016. Patients whose date of diagnosis to date of death was 3 years or less were selected as this population would be more likely to be enriched for *CCNE1* amplification. Patients who fit these criteria were further screened for presence of metastatic sites of disease with available pathology specimens. Those that met inclusion criteria were selected for screening for *CCNE1* amplification through ddPCR.

### 2.2 DNA extraction from FFPE tissue

DNA was extracted from FFPE tissue from each metastatic site using standard laboratory protocols. Representative slides stained with hematoxylin and eosin (H&E) were used to identify areas with at least 50% tumor cell nuclei to ensure adequate tumor purity. Normal tissue specimens from select samples were extracted to serve as diploid controls. DNA extraction was performed using the DNeasy Blood and Tissue Kit (Qiagen) and quantified using the Qubit dsDNA High Sensitivity Assay Kit (Thermo Fisher) prior to use in downstream applications.

### 2.3 Copy number analysis by digital droplet PCR (ddPCR)

Copy number of *CCNE1* in the extracted DNA from FFPE specimens was detected using QX200 Droplet Digital PCR System (Bio-Rad), which provides absolute quantification of DNA content with high precision (±10%). Primers and probes for *CCNE1* (targeting region) and for ribonuclease P/MRP 30 kDa subunit (control; targeting region) were obtained from Bio-Rad (*CCNE1* probe dHsaCP2500372, RPP30 probe dHSACP 2500350). For each reaction, 10 ng of restriction digested DNA was mixed with 1X ddPCR Supermix for probes (Bio-Rad) and primer probes for both *CCNE1* (FAM probe) & RPP30 (Hex probe) to a final volume of 20 μL, as recommended by the manufacturer. Reactions without any DNA template were also run as negative controls on every PCR plate. Following emulsion generation on the QX200 Automated Droplet Generator (Bio-Rad), the samples in a 96-well PCR plate were heat-sealed with foil, and amplified in a thermal cycler for 40 cycles with an annealing temperature of 58 deg Celsius. Post PCR, the droplets were read using QX200 Droplet reader (Bio-Rad) and CNVs were determined using the QuantaSoft™ Software version 1.7 (Bio-Rad). For this study, amplification was defined as copy number > 5, gain was defined as copy number from 3 to 5, and normal (diploid) was defined as copy number of less than 3. Samples with known copy number status from Foundation Medicine testing were used as positive and negative controls for assay validation.

### 2.4 Tumor purity assessment by immunohistochemistry and by TP53 sequencing

Prior to DNA extraction, representative H&E slides from each sample were reviewed with a pathologist to assess the estimated percentage of HGSC tumor cell nuclei within the region selected for extraction. Next-generation sequencing of *TP53* was also completed to confirm tumor purity, as described below. The same DNA samples that were used for copy number analysis were used for sequencing whenever possible. Libraries were prepared with a custom AmpliSeq for Illumina panel targeting the coding region of *TP53*. Quality control analysis of libraries was completed with the Agilent High Sensitivity D1000 ScreenTape System prior to sequencing on the MiniSeq platform. Bioinformatic analysis was completed using the DNA Amplicon pipeline in the Illumina BaseSpace Sequence Hub, including alignment with BWA and variant calling by Illumina’s somatic variant caller. Subsequent variant annotation was performed using wAnnovar. Identified variants were filtered and manually reviewed in the integrative genomics viewer.

### 2.5 Data analysis

A correction for tumor purity was used in the calculation of *CCNE1* amplification using the digital droplet PCR values based on prior work. (Carter et al., 2012) Since *CCNE1* amplification is a somatic event and cancer samples are a combination of normal and tumor tissues, any normal tissue contamination will reduce the true quantification of *CCNE1* amplification proportionally. Tumor purity was calculated using the following formula: (ddPCR *CCNE1* copy number-(2*(1-Tumor purity)))/Tumor purity. Calculations were performed using both *TP53* allele frequency from sequencing data and percent tumor purity from H&E samples and *TP53* allele fraction calculations were preferentially used. GraphPad Prism (version 8.2.0) was used to generate graphical representations of *CCNE1* amplification.

## 3 Results

**Fig. 1:**
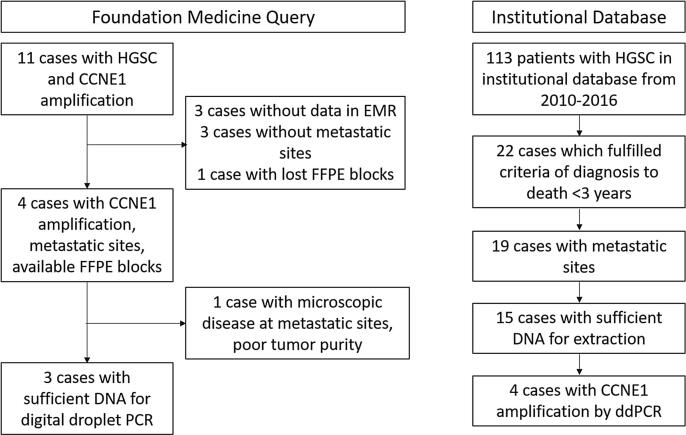
Selection of cases by foundation medicine query and digital droplet PCR Screening of cases from an institutional database. HGSC, high grade serous cancer; FFPE, formalin fixed paraffin embedded; PCR, polymerase chain reaction; ddPCR, digital droplet polymerase chain reaction.

Of the 15 HGSC cases identified from our institutional database for *CCNE1* ddPCR screening, four (27%) were found to have *CCNE1* amplification. Four additional cases were included from commercial clinical testing results, one of which subsequently had insufficient tumor content for further analysis. These seven cases of HGSC with confirmed *CCNE1* amplification in the primary tumor had specimens from metastatic sites available for inclusion in this study (Fig. 1) and represented ovarian (n = 2), uterine (n = 2) and fallopian tube carcinomas (n = 3) (Table 1). Each complete patient case included two to five metastatic disease sites for evaluation of *CCNE1* copy number yielding 18 total metastatic sites for testing. Multiple samples were taken from each anatomic site when sufficient tumor size permitted. Four samples that were screened with ddPCR and found to be diploid were chosen as negative controls. Of 73 total samples evaluated by ddPCR, 68 had sufficient DNA for *TP53* sequencing. Average coverage of the targeted region across all sequenced samples was 7158X.

**Table 1:** Summary of clinical information for identified *CCNE1* amplified cases. BM samples: Samples identified from foundation medicine query; SCR samples: samples identified by digital droplet PCR.

Sample Name	Surgery Completed	Histology	Metastatic sites
BM6	Exploratory laparotomy, total abdominal hysterectomy, bilateral salpingo oophorectomy, pelvic and para aortic lymph node dissection	Uterine serous adenocarcinoma	Uterus, Pelvic lymph node (2), Para aortic lymph node (2)
BM7	Exploratory laparotomy, total abdominal hysterectomy, bilateral salpingo oophorectomy, pelvic and para aortic lymph node dissection, tumor debulking	Ovarian high-grade serous carcinoma	Left adnexa, Right adnexa, Cul de sac nodule
BM8	Robot assisted total laparoscopic hysterectomy, bilateral salpingo oophorectomy, pelvic lymph node dissection, omentectomy	Uterine serous adenocarcinoma	Uterus, Pelvic lymph node
SCR3	Exploratory laparotomy, total abdominal hysterectomy, bilateral salpingo oophorectomy, omentectomy, tumor debulking, intraperitoneal port insertion	Fallopian tube high grade serous carcinoma	Left ovary, Right ovary
SCR5	Exploratory laparotomy, total abdominal hysterectomy, bilateral salpingo oophorectomy, omentectomy, resection of lesser sac mass, tumor debulking, intraperitoneal port insertion	Fallopian tube high grade serous carcinoma	Right ovary, left fallopian tube, omentum, lesser sac nodule
SCR6	Exploratory laparotomy, total abdominal hysterectomy, bilateral salpingo oophorectomy, omentectomy, transverse colectomy, tumor debulking	Fallopian tube high grade serous carcinoma	Left adnexa, round ligament nodule, transverse colon, right adnexa
SCR10	Exploratory laparotomy, bilateral salpingo oophorectomy, omentectomy, rectosigmoid resection, tumor debulking	Ovarian carcinosarcoma (70% high-grade serous component)	Omentum, splenic flexure tumor, right adnexa, peritoneal implant

**Fig. 2:**
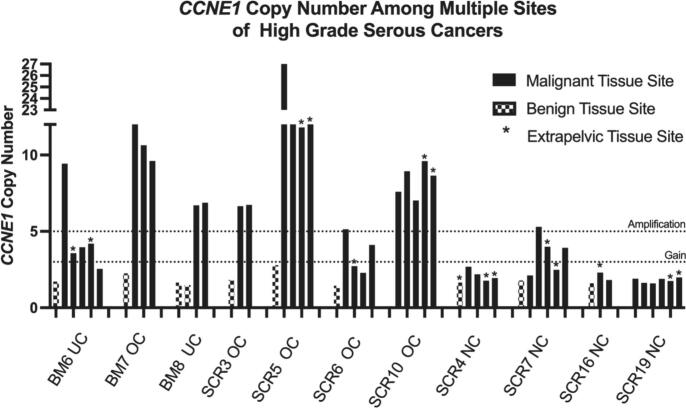
*CCNE1* Copy number determined by digital droplet PCR for multiple Sites of patients with known *CCNE1* amplified and diploid high grade serous tumors. UC, uterine cancer; OC, ovarian cancer; NC, negative control (Diploid *CCNE1*).

**Fig. 3:**
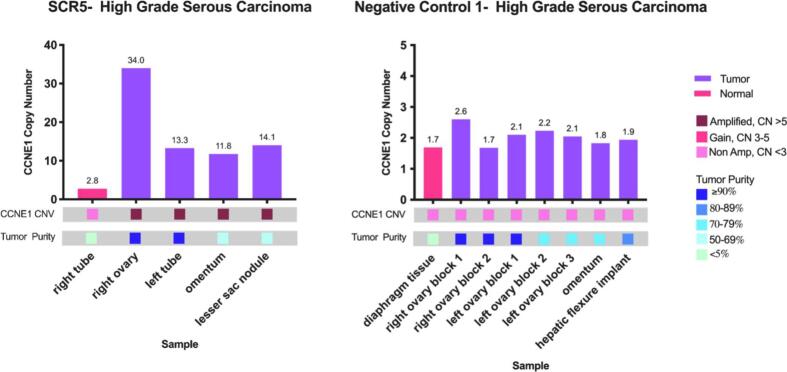
Examples of *CCNE1* copy number from metastatic sites among amplified and control cases. CN, copy number; CNV, copy number variation.

Of the seven cases with *CCNE1* amplification, five cases (71%) showed uniform amplification of *CCNE1* across all tested metastatic sites (Fig. 3). Two cases (BM6, SCR6) showed heterogeneity with most metastatic sites showing *CCNE1* copy number gain rather than amplification. In both of these cases the initially screened sample showed *CCNE1* amplification and the remaining tested sites showed copy number gain or a near diploid copy number. Though amplification may change during the metastatic process, consistency among metastatic sites suggests that this process is does not continue to evolve. Among patients who displayed *CCNE1* amplification among all metastatic sites (BM7, SCR5, SCR10), there was heterogeneity in the absolute *CCNE1* copy number between metastatic sites. Three of the four negative control cases had uniform diploid copy number across all metastatic sites. One control case showed some heterogeneity across various metastatic sites showing (Fig. 2). *CCNE1* copy number of non-tumor internal controls across all samples were diploid. Detailed representations of *CCNE1*-amplified tumor and normal sites along with their sample tumor purity are shown in Fig. 3.

## 4 Discussion

In this group of HGSC cases with known *CCNE1* amplification, amplification largely appears conserved across metastatic sites of disease consistent with its presumed role as a truncal driver event, based on the occurrence of CCNE1 amplification in ovarian cancer precursor lesions. (Karst et al., 2014, Kuhn et al., 2016) The population of *CCNE1* amplified gynecologic HGSC represents an unmet need for molecularly driven treatment and thus far no therapeutics have entered into routine clinical practice for this specific population. Pre-clinical data support targeting of the Cyclin E pathway for therapeutic benefit. Knockdown of the *CCNE1* and CDK2 genes in known *CCNE1* amplified ovarian cancer cell lines has led to reduced clonogenic survival, but this has not been seen with the CDK2 inhibitor daniciclib when given as monotherapy. (Au-Yeung et al., 2017) More than 20 CDK inhibitors exist and have been tested on *CCNE1* amplified ovarian cancer cell lines with inconsistent efficacy and proposed resistance mechanisms including an increase in pro-survival signaling. (Etemadmoghadam et al., 2013) There is evidence that redundancy and compensatory pathways in the G1-S transition can account for the failure of CDK2 inhibition alone to achieve cell cycle arrest. (Aleem et al., 2005) Dinaciclib in combination with platinum based chemotherapy agents has shown activity in OVCAR3 mouse xenograft models suggesting that CDK2 inhibition can be paired with other therapies to achieve activity in *CCNE1* amplified HGSC. (Taylor-Harding et al., 2015)

As the evidence for *CCNE1*′s role in the molecular characterization of HGSC strengthens, more effort can be directed in assessing a therapeutic to target its molecular action. Despite the failure of CDK inhibitors to emerge as a clinically active treatment option, there are other biologically plausible functions of *CCNE1* that could be targeted including its impact on DNA replication, DNA repair, apoptosis, cell cycle regulation, DNA transcription and centrosome amplification. (Kanska et al., 2016) *CCNE1* amplification’s impact on rendering cells sensitive to replication stress has been tested in a phase II randomized trial of gemcitabine with or without the ATR inhibitor berzosertib. The most benefit was seen in patients with a platinum free interval of less than three months, which is thought to represent an enriched *CCNE1* amplified population. (Konstantinopoulos et al., 2020) Given *CCNE1* amplification and BRCA1/2 mutations are mutually exclusive, there is thought that *CCNE1* amplified cancers are reliant on a proficient homologous recombination pathway. (Etemadmoghadam et al., 2013) Early clinical data of the combination of checkpoint kinase inhibition and immune checkpoint blockade showed durable responses in several CCNE1 amplified patients with high grade serous ovarian cancers, suggesting an interplay between cell cycle inhibition, DNA damage repair and the immune response. (Do et al., 2021) Our data that *CCNE1* amplification remains present in the metastatic disease sites provides support that molecularly targeted approaches could be therapeutically active in advanced disease.

In this study, we used an inclusion criterion of poor survival to enrich our screened samples for *CCNE1* amplification and achieved a *CCNE1* amplification prevalence of 27%. This is consistent with the expected frequency of *CCNE1* amplification seen in the overall HGSC population (~20%) and confirms that *CCNE1* amplification is more common in poor outcome situations. Given that we used two methods to infer tumor purity (targeted sequencing and H&E evaluation), it is unlikely that we missed *CCNE1*-amplified cases due to low tumor content in DNA samples. Our control methods of using non-tumor tissue from *CCNE1*-amplified cases as well as non-*CCNE1*-amplified ovarian cancer cases helps to correctly identified *CCNE1* amplification. We had attempted to quantify cyclin E expression with immunohistochemistry (IHC), but contrary to previous reports, (Karst et al., 2014, Nakayama et al., 2010, Ayhan et al., 2017, Goundiam et al., 2015) we did not find IHC useful due to variable staining patterns in both amplified and diploid cases, likely due to variability in commercially available antibodies limiting sensitivity, precision, and reproducibility. This study is limited by sample selection bias. We used a sample of convenience based on available commercial molecular profiling and tissue availability. These limitations would bias the case series toward those cases that are more advanced at presentation (i.e., adequate tumor content) and with a propensity for recurrence (i.e., those having molecular profiling).

## 5 Conclusion

*CCNE1* amplification in HGSC is a molecularly conserved event that is present across metastatic sites and is thought to be a driver event. Limited heterogeneity in *CCNE1* copy number among *CCNE1*-amplified cases suggests minimal ongoing genomic change during the metastasis. Successful targeting of the downstream effects of *CCNE1* amplification will provide a much-needed therapeutic opportunity to this population of patients with limited effective treatment options.

## 6 Sources of funding

This work is supported in part by 10.13039/100000005US Department of Defense Award: W81XWH-15-1-0429, NIH:P30 CA016087, The V Foundation for Cancer Research, The Honorable Tina Brozman Foundation for Ovarian Cancer Research, and Arnold Chavkin and Laura Chang.

## Declaration of Competing Interest

The authors declare that they have no known competing financial interests or personal relationships that could have appeared to influence the work reported in this paper.
